# Heart Murmur Detection in Phonocardiogram Data Leveraging Data Augmentation and Artificial Intelligence

**DOI:** 10.3390/diagnostics15192471

**Published:** 2025-09-27

**Authors:** Melissa Valaee, Shahram Shirani

**Affiliations:** 1 Faculty of Health Sciences, McMaster University, Hamilton, ON L8N 3Z5, Canada; 2 Department of Electrical & Computer Engineering, Faculty of Engineering, McMaster University, Hamilton, ON L8S 4K1, Canada; shirani@mcmaster.ca

**Keywords:** heart murmur detection, heart valves, artificial intelligence, machine learning, phonocardiogram

## Abstract

**Background/Objectives**: With a 17.9 million annual mortality rate, cardiovascular disease is the leading global cause of death. As such, early detection and disease diagnosis are critical for effective treatment and symptom management. Cardiac auscultation, the process of listening to the heartbeat, often provides the first indication of underlying cardiac conditions. This practice allows for the identification of heart murmurs caused by turbulent blood flow. In this exploratory research paper, we propose an AI model to streamline this process to improve diagnostic accuracy and efficiency. **Methods**: We utilized data from the 2022 George Moody PhysioNet Heart Sound Classification Challenge, comprising phonocardiogram recordings of individuals under 21 years of age in Northeast Brazil. Only patients who had recordings from all four heart valves were included in our dataset. Audio files were synchronized across all recordings and converted to Mel spectrograms before being passed into a pre-trained Vision Transformer, and finally a MiniROCKET model. Additionally, data augmentation was conducted on audio files and spectrograms to generate new data, extending our total sample size from 928 spectrograms to 14,848. **Results**: Compared to the existing methods in the literature, our model yielded significantly enhanced quality assessment metrics, including Weighted Accuracy, Sensitivity, and F-Score, and resulted in a fast evaluation speed of 0.02 s per patient. **Conclusions**: The implementation of our method for the detection of heart murmurs can supplement physician diagnosis and contribute to earlier detection of underlying cardiovascular conditions, fast diagnosis times, increased scalability, and enhanced adaptability.

## 1. Introduction

With an estimated 17.9 million deaths per year, cardiovascular diseases are the leading cause of death globally [1]. Early detection of such diseases is critical in their treatment and management. One critical diagnostic test for the detection of underlying cardiac conditions is cardiac auscultation. This practice involves listening to the heartbeat to identify heart murmurs caused by turbulent blood flow, which may be indicative of underlying complications [2]. Auscultation is conducted in routine physical examination and is traditionally carried out with a stethoscope [2,3]. Unfortunately, this method has limited sensitivity and accuracy due to high inter-rater discrepancy, even among trained healthcare professionals [3]. Given the importance of heart murmur detection in the diagnostic process, an enhanced technique for murmur detection is necessary.

In recent years, evidence-based research regarding the applications of artificial intelligence in the medical field has demonstrated increased promise as a way to streamline and supplement physician diagnosis. Artificial intelligence has been implemented in various medical settings to enhance patient care through improved diagnostic capabilities, streamlined workflow and reduced medical errors and costs [4]. However, artificial intelligence, also limited in its efficacy, is heavily reliant on the data used to train the model. To ensure efficacy, algorithms must be trained on a large volume of high-quality data. Outdated, imbalanced, or biased data may result in severe compromises [5].

To address the above limitations in cardiac auscultation, we propose an artificial intelligence model that can detect heart murmurs with high accuracy and speed. Our model, leveraging data augmentation and machine learning, demonstrates increased Weighted Accuracy, Sensitivity, Specificity, Negative Predictive Value, Positive Predictive Value, and F-Score compared to the pre-existing methods, while allowing for faster processing and classification. The implementation of our method may result in fewer false positive and false negative tests, which promotes the appropriate allocation of medical resources, ensuring all patients with cardiac conditions receive appropriate care without subjecting healthy individuals to unnecessary investigations.

## 2. Materials and Methods

**Figure 1:**
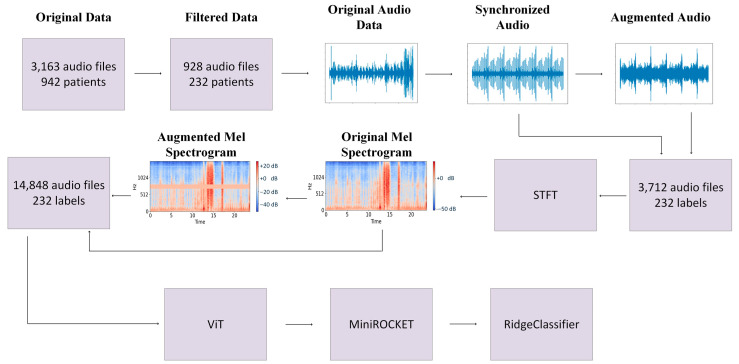
A visualization of our methodology, beginning with original sound data, which was then filtered such that only patients with data from all four heart valves were used. The heartbeats are then temporally aligned, and various augmentation techniques are implemented to generate more audio data. Then, a Mel spectrogram is generated from all data (real and augmented). Subsequently, a number of augmented spectrograms are generated from the original spectrogram. Each Mel spectrogram is then passed through the Vision Transformer, which generates the logit vector that is passed to the MiniROCKET model. Finally, a Ridge Classifier is used for classification.

**Figure 2:**
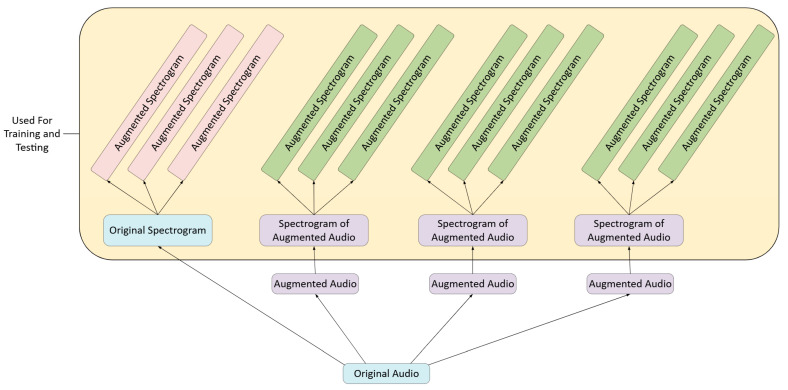
A visualization of the Data Augmentation process. Each original PCG audio file yielded three augmented audio files. From each audio file, one Mel spectrogram was generated, which served as the template for three additional augmented spectrograms. The color of the boxes indicates where the audio file or spectrogram is sourced, with blue representing original audio files and their corresponding spectrograms, purple representing spectrograms generated from augmented audio files, pink representing augmented spectrograms from spectrograms of original audio, and green representing augmented spectrograms generated from spectrograms of augmented audio.

Our proposed heart murmur detection method, as outlined in Figure 1, uses Multivariate Minimally Random Convolutional Kernel Transform (MiniROCKET) as its foundation [6]. Phonocardiogram data was obtained from the CirCor DigiScope Dataset from the George B. Moody 2022 PhysioNet Heart Sound Classification Challenge Database, a challenge organized by PhysioNet (maintained by researchers at the Massachusetts Institute of Technology) and the Computing in Cardiology Conference. The dataset was filtered to include only trials that had recordings of all four valves [7,8]. Each set of four phonocardiogram recordings underwent a series of 10 random time-series data augmentation techniques applied a total of four times. Subsequently, a Mel spectrogram was generated from each audio file. For each of these spectrograms, one random data augmentation technique is applied three times to generate a total of three augmented spectrograms (Figure 2). All spectrograms are then flattened and passed into a pre-trained Vision Transformer, and a vector of logits is returned. Then, a set of fixed kernels is convolved with the logit vector. The result is then shifted by a random amount, and the frequency of the number of times the resulting trace becomes positive is represented as a feature map that is input to a ridge regressor to complete the classification.

### 2.1. Data Availability

All data used throughout the training and testing phases of our model development were obtained from the CirCor DigiScope Phonocardiogram Dataset for the George B. Moody PhysioNet Challenge 2022 on Heart Murmur Detection from Phonocardiogram Recordings [7]. This publicly available dataset contains 5272 sound recordings from 1568 anonymized patients aged 0 to 21 years collected in Northeast Brazil from July to August of 2014 and June to July of 2015. The dataset contains recordings from all primary auscultation points: the Aortic Valve, the Pulmonic Valve, the Tricuspid Valve, and the Mitral Valve; however, not all patients have recordings for all four valves. Furthermore, each audio file was labeled based on the presence or absence of a murmur in the trace. We included only patients who had data from all four valves, grouping them and assigning a label based on whether a murmur was present at any of the four valves. Thus, we began with a sample size of 928 audio files spanning 232 labels (each labeled patient consisted of four audio recordings, one for each valve).

### 2.2. Audio Synchronization

In our methodology, we leverage Multivariate MiniROCKET, a supervised machine learning algorithm designed to rapidly and accurately classify data based on multiple directly correlated input channels [6]. For our implementation, audio from each valve is considered its own channel, where the channels are made congruous through heartbeat synchronization. At a given time, two audio signals are compared to each other; the heartbeat peaks are identified and lined up such that the first heartbeat of each audio file occurs at the same timestamp. This process is carried out until all four audio signals are aligned with each other. Then, each signal is looped until it reaches a time of 58 s—the length of our longest audio file—to ensure that all model inputs are the same length.

### 2.3. Data Augmentation

#### 2.3.1. Audio File Augmentation

Data augmentation is a process that generates new samples from existing data [9]. This task is accomplished by applying various transformations to the original dataset, such as rotation, flipping, and noise injection. In applying these transformations, the model is able to learn from a more diverse set of inputs, thus increasing its robustness and generalizability.

Data augmentation is particularly beneficial in situations where collecting labeled data is challenging, expensive, or time-consuming [10]. Through data augmentation, the dataset can be expanded, which allows the model to more easily and accurately identify patterns in data, and thus, yields improved accuracy when exposed to new data. Additionally, data augmentation promotes data diversity; thus, the model may learn to better handle variations in data [11].

Furthermore, data augmentation helps prevent overfitting of the model. Overfitting occurs when a model simply memorizes the training data rather than identifying the overarching patterns. In these instances, the model performs poorly on unseen data. However, introducing variations in the training data through data augmentation forces the model to learn generalized features, thus reducing the risk of overfitting [9,11].

**Figure 3:**
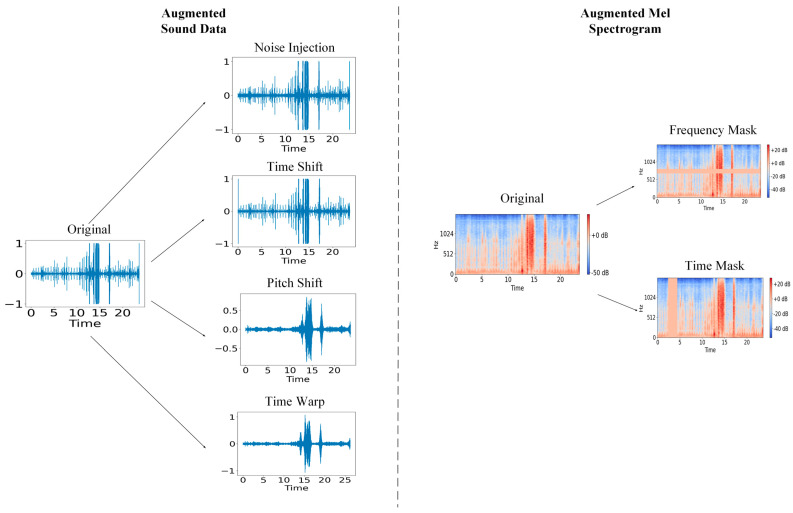
A visualization of the implemented data augmentation techniques. For sound data (**left**), we implemented noise injection, time shift, pitch shift, and time warp. For Mel spectrograms (**right**), we implemented frequency masking and time masking.

We implemented various data augmentation techniques at two distinct stages in our methodology. We based our augmentation techniques on those commonly used in existing literature [9]. We applied ten random transformations from the following list to each audio file: Gaussian noise injection, pitch shift, time shift, and time warp (Figure 3).

*Gaussian Noise Injection*: Gaussian Noise, common in machine learning applications [9], is a type of random variable that follows a bell-shaped normal distribution, with uncorrelated samples. First, a sequence of random numbers is generated such that the length of this sequence is equal to the length of the input data, the mean of the sequence is 0, and its standard deviation is 1. It is then scaled by a noise factor and added to the input data in an element-wise fashion. Thus, each element in the noise sequence is summed with the corresponding index of the data signal sequence.

*Pitch Shift*: Pitch refers to the perceived frequency of a sound, with higher pitch sounds corresponding to higher frequency. First, the spectrogram, containing the time-frequency representation of the audio file, is generated. Then, pitch shifting is completed by manipulating the frequencies according to the following formula

$$X^{\prime }\left(t,\;\omega \right)=X\left(t,\;\alpha \omega \right)$$

where *X(t*, *ω*) is the original frequency representation, *α* is the pitch-shifting factor (*α* > 1 increases the pitch), and *X*′(*t*, *ω*) is the pitch-shifted frequency representation. During this process, all frequency components are shifted together (both the fundamental frequency and all the associated harmonics), thus preserving the sound of the original data. Finally, the audio signal is reconstructed from the augmented spectrogram. This technique was selected for its ability to simulate anatomical variations in the heart, particularly those involving heart size and myocardium thickness.

*Time Shift*: Time Shift causes a randomly generated unidirectional shift in the data. This is achieved by shifting the values in each index of the data signal. The order of the data is preserved, but each point is moved to a different timestamp. In our implementation, the values at the ends of the signal are wrapped around at the boundary to preserve the signal upon shifting. This technique was utilized to allow the model to detect murmurs regardless of where they appear in the signal.

*Time Warping*: Time Warping alters the temporal characteristics of the audio file while preserving the original pitch. To achieve this, the audio file is first converted to the time-frequency domain by a Short Time Fourier Transform (STFT). An STFT converts the audio signal into a spectrogram, which is a visual representation of the audio signal’s frequency over time. In this representation, the time domain can be manipulated independently of pitch. Finally, the audio file is restored to its initial representation through an inverse STFT. Time warping served to model variations in heart rate as well as the duration of the cardiac cycle among patients.

All transformations were applied across all four data channels to preserve their correlation. From each patient’s original data, we generated three sets of augmented audio data, bringing our total number of sound recordings to 3712 audio files from our original 928 files.

#### 2.3.2. Mel Spectrogram Augmentation

Subsequently, a Mel spectrogram is utilized to more closely mimic human auditory perception. To achieve this representation, each audio file is converted from the time-decibel domain to the time-frequency domain. Then, the existing Mel filter bank is applied. This bank is a series of triangular filters spaced to best mimic human processing, with lower frequencies that have a higher resolution, represented more linearly, and higher frequencies with a lower resolution represented more logarithmically. The resulting matrix is then normalized to reduce the computational cost in subsequent processing.

To further increase the robustness of our model, we also applied data augmentation techniques to the spectrograms. We randomly selected either a frequency mask or a time mask (Figure 3). Then, we set the value of a randomly selected point in the spectrogram to 0 for a duration that was randomly selected between 0 and 20 ms. For a frequency mask, a particular range of frequencies in the spectrogram is set to 0 for the duration of the spectrogram. For the time mask, all the frequences for a particular time range are set to 0. In total, from each audio file (original and augmented), we generated one non-augmented spectrogram and three augmented ones [12]. Thus, we had a total of 14,848 spectrograms, corresponding to 3712 labeled groups, since there were 4 recordings per group.

### 2.4. Algorithm Development

#### 2.4.1. Vision Transformer

**Figure 4:**
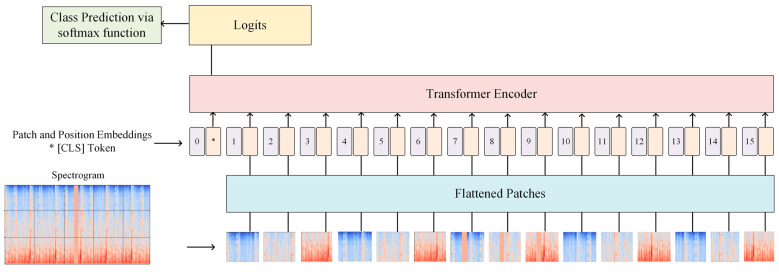
A visualization of the Vision Transformer methodology, beginning with patching and subsequent linear projection. Then, positional encoding is conducted, and the [CLS] token is added at the location marked with *, before inputting to the transformer. The transformer outputs a vector of logits that will be used for class prediction.

A Vision Transformer (ViT) is a deep-learning method that is effective in image recognition [13] (Figure 4). A notable feature of ViT is its patch-like approach to image classification. First, the input image is divided into patches of 16 × 16 pixels. Each patch is subsequently flattened into a 1-dimensional vector and is added a unique identifier to preserve the spatial orientation of the patch (called positional encoding). The patches with their respective positional encoding are passed to a transformer network that utilizes a self-attention mechanism to assess and learn how the patches are related. During this process, a specialized token, called the [CLS] token, is added to the beginning of patch embedding sequences to gather information from all patches. Once transformer processing is complete, the [CLS] token will contain all critical information from the image. The [CLS] token is then passed to a dense neural network layer of the Transform Encoder, which will calculate a score for each preexisting category. These scores are termed logits and are still considered raw, unnormalized values. Conventionally, logits would undergo further processing to allow for classification of the image; however, we implemented ViT as an additional measure for image pre-processing, thus this step is bypassed.

#### 2.4.2. MiniROCKET

The returned logits are then stored in a 2-dimensional structure similar to a table, called a DataFrame. This DataFrame contains two columns, one for the file name and one for the logit values. There is one row for each audio file. This data is then stored and reshaped into a 3-dimensional NumPy array called the X array. This array has dimensions (labeled groups, audio files, logits); thus, its shape is (3712, 4, 1000). The ground truth diagnosis for each patient, either ‘murmur present’ or ‘murmur absent,’ is stored in a corresponding Y array, such that the diagnosis and data will be at the same index in their respective arrays.

The indices of the arrays are then randomly shuffled while preserving the X array and Y array compatibility. The data is then split into a training and testing dataset, with 80% of samples selected for the training set while the remaining 20% become the testing set. The training and testing sets contained both the original and the augmented data.

**Figure 5:**
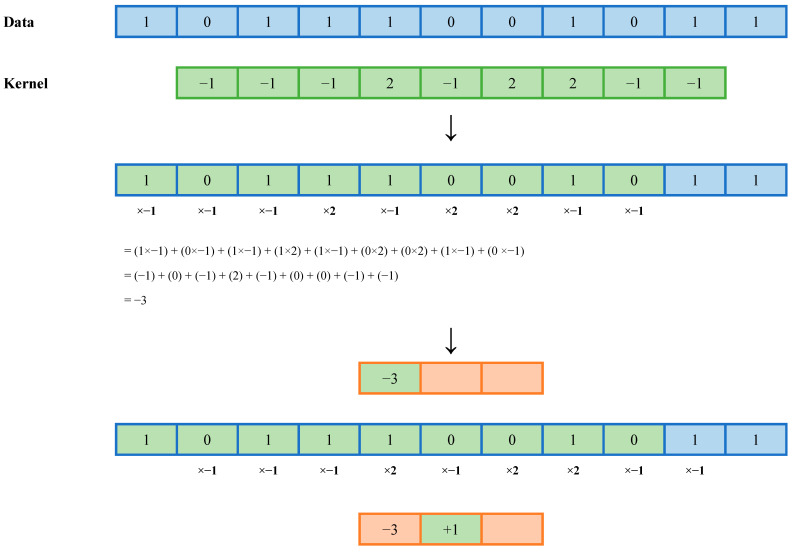
A visualization of dot product calculation through kernel convolution with a kernel size of 3 × 3 and a dilation rate of 1. The dot product is calculated as the sum of the products of each data pixel and the value of the corresponding kernel pixel. The kernel passes over the data to read it all in this fashion. The blue coloring and outline represents the data vector, the green coloring represent the current location of the kernel, and the orange represents the feature map.

MiniROCKET uses a 1 × 9 matrix, called a kernel [6]. Each pixel in the kernel is assigned a weight of either −1 or +2 such that the sum of the kernel weights is equal to zero [6]. Each kernel is then passed over the data to calculate the convolution and generate a new signal of similar length to the original data (Figure 5). In a multivariate model, such as ours, each kernel is passed over each channel, corresponding to each heart valve, individually. Subsequently, the data are concatenated [6].

This process is completed with 10,000 distinct kernels to generate a feature map. This feature map then serves as the input for a Ridge Classifier, a specialized linear regression model that has been enhanced to prevent overfitting and increase generalizability during the classification process [14]. Additionally, we considered implementing a buffering range to increase confidence in the model’s classification. However, after empirically assessing the model’s accuracy and considering the coverage tradeoff, we determined that the classifier’s traditional ‘0’ threshold and zero buffering provided the highest accuracy.

### 2.5. Statistical Analysis

Once training and testing were completed, we assessed three aspects of our methodology:

1. The effect of varying levels of data augmentation
2. The overall accuracy of our MiniROCKET methodology
3. The speed of our model

This assessment was conducted by using numerous metrics to draw comparisons between our model and pre-existing models. Notably, we calculate Sensitivity, Specificity, Positive Predictive Value, Negative Predictive Value, F-Score, Accuracy and Weighted Accuracy. We also assessed the evaluation time of our model.

In our assessment, “True Positive” is synonymous with “Truly Present” and “True Negative” is synonymous with “Truly Absent”.

*F-Score*: The F-Score provides an overall assessment of the algorithm’s functioning for each class (“present” or “absent”) with respect to Positive Predictive Value and Sensitivity, and is represented by the following formula [15]

$$F\text{-}Score=2\times \frac{Positive\;Predictive\;Value\times Sensitivity}{Positive\;Predictive\;Value+Sensitivity}$$

*Weighted Accuracy*: Weighted Accuracy (WAcc) is a metric defined by the PhysioNet Challenge, which assigns a weight of 5 to True Positives to increase their significance in the accuracy calculation. It is represented by the following formula

$$WAcc=\frac{5\left(True\;Positives\right)+(True\;Negatives)}{5\left(True\;Positives+False\;Negatives\right)+(False\;Positives+True\;Negatives)}$$

## 3. Results

### 3.1. Assessment of Vision Transformer

We first assessed the performance of our model with and without the implementation of the pre-trained Vision Transformer. The results in Table 1 were obtained.

As depicted in Table 1, the accuracy of the classification remained stable with the introduction of the ViT, while the time taken to evaluate each spectrogram was significantly reduced from 50.405 s to 0.019 s. The Vision Transformer’s ability to condense the data from the spectrogram into a smaller vector, allows for faster computation by the MiniROCKET-Ridge Classifier pipeline.

### 3.2. Assessment of Data Augmentation Techniques

**Figure 6:**
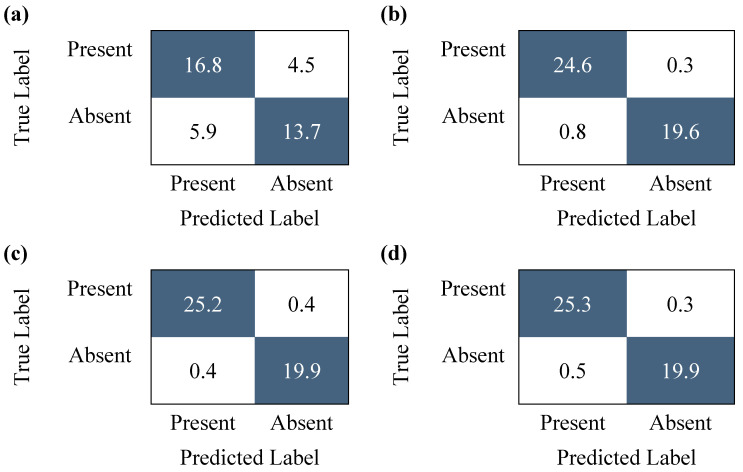
The confusion matrices were generated during testing phases with various quantities of augmented data. Values in blue were correctly classified. (**a**) The confusion matrix generated when no augmented data was used. (**b**) The confusion matrix generated from original audio data and 1 augmented audio file, then 1 non-augmented spectrogram and 1 augmented spectrogram from each original audio file for a total of 3712 files. (**c**) The confusion matrix generated when 2 augmented audio files and 2 augmented spectrograms were generated from each original audio file for a total of 8352 files. (**d**) The confusion matrix generated when 3 augmented audio files and 3 augmented spectrograms were generated from each original audio file for a total of 14,848 files.

Once training and testing were completed, the Confusion Matrices in Figure 6 were obtained. Each matrix was generated with increasing amounts of augmented training data and outlines the average values obtained after 10 tests. Subsequently, we conducted a quality metric analysis for each matrix to observe how these metrics change as the sample size is increased. An Analysis of Variance (ANOVA) was used to assess the statistical significance of the observed improvements. Our statistical results are outlined in Table 2 and Table 3.

The results of the ANOVA yielded a statistically significant improvement in all calculated metrics. Subsequently, Tukey’s Honestly Significant Difference was used to identify which specific groups have significant differences between them. The results, as presented in Table 2, demonstrate that all significant differences were observed in all comparisons except between the Medium and Heavy Augmentation groups. Thus, increasing the levels of augmentation contributes to the overall enhancement of the classification model; however, this results in a plateau at a certain point.

However, since the testing dataset was kept consistent across all levels of augmentation, the assessment of Heavy Augmentation is not fully valid as the testing set was too small relative to the training set. Thus, we conducted an appropriate assessment of the method with a conventional 80%/20% training/testing split, the results of which are demonstrated in Table 4.

### 3.3. Comparison with Pre-Existing Models

To assess the applicability of our model, we compare our metrics to pre-existing methodologies, notably the top 5 algorithms from the original 2022 PhysioNet Challenge, as well as the method introduced by Manshadi & Mihandoost in 2024 [16].

Manshadi & Mihandoost introduce a semi-supervised model for heart murmur detection in phonocardiogram data. Like us, they utilize data from the 2022 CirCor DigiScope Phonocardiogram Dataset. Their method begins by conducting a Stockwell transform to convert phonocardiogram data to 2-dimensional time-frequency maps [17]. Then, a deep convolutional neural network called AlexNet is used to extract deep features before subsequently passing them into a Support Vector Machine (SVM) Classifier. The metrics for their study are presented in Table 5, alongside the results of the top 5 algorithms from the original 2022 PhysioNet Challenge.

As depicted, our quality metric assessments demonstrate an enhanced murmur detection method when compared to all 2022 PhysioNet Challenge algorithms as well as the one proposed by Manshadi & Mihandoost [16].

### 3.4. Evaluation Time Assessment

A comparison with the PhysioNet Challenge algorithms highlighted our model’s enhanced efficiency, with our model spending roughly 0.02 s per patient during the evaluation phase, while the most efficient PhysioNet Challenge model, proposed by Xu et al., spent 0.24 s per patient [19]. Thus, our method is considerably faster.

With a Weighted Accuracy of 96.4%, Sensitivity of 96.6%, and F-Score of 0.960, as well as an evaluation time of 0.02 s per patient, our MiniROCKET implementation is superior compared to the pre-existing methods.

### 3.5. Feature Importance Assessment

Finally, we explored which features contribute most to the RidgeClassifier’s classification. Features corresponding to smaller values of the classifier weights will have a negligible impact on the outcome of the classification.

**Figure 7:**
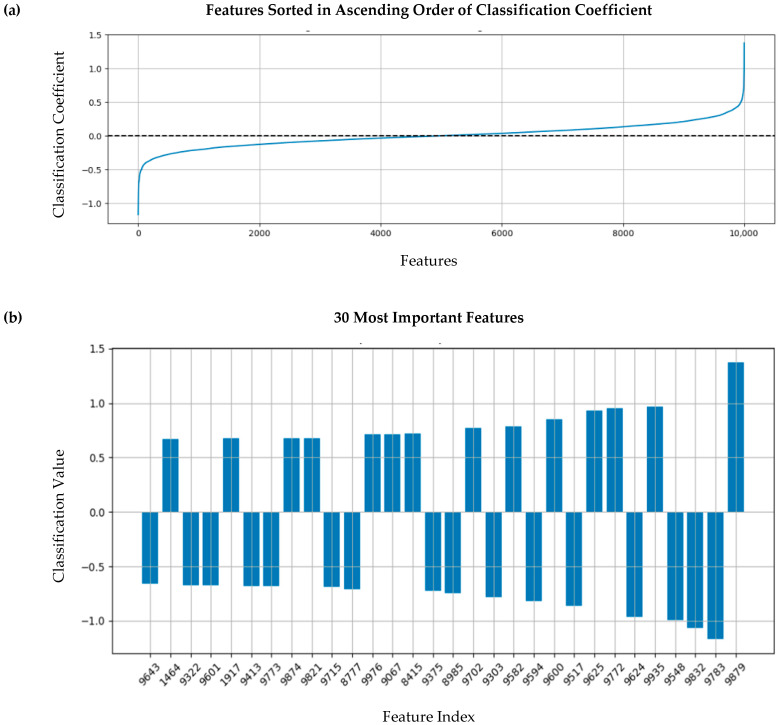
A visualization of the degree of importance for different features. (**a**) A visualization of the features in ascending order to highlight the distribution of the classification coefficients. (**b**) A presentation of the 30 most important features for the classification.

Figure 7a illustrates the classifier coefficients in increasing order. As noticed, a large range of coefficients fall within a small neighborhood of 0. Multiplication of these coefficients by their corresponding features nullifies the effect of those features, rendering them less important. In this figure, approximately 4000 features, located in the ranges of 0 to 2000 and 8000 to 10,000, contribute significantly to the classification. Figure 7b illustrates the top 30 important features alongside their coefficients and index number.

## 4. Discussion

MiniROCKET provides several unique advantages. First, it has fewer trainable parameters compared to other machine learning methods, thus rendering it effective—particularly for smaller datasets—enabling faster classification. Next, by using 10,000 convolution kernels, MiniROCKET strikes a balance between high accuracy and reduced computational costs, thus boasting improved classification efficiency [6]. Additionally, the utilization of random kernels prevents over-training and allows the model to focus on broad patterns in the data. Thus, it can effectively handle noise, making it especially robust for real-world applications [6]. Our results are further improved by leveraging data augmentation techniques to increase the dataset size. The advantages of these techniques are evidenced by the results of our model. With a Weighted Accuracy of 96.4%, Sensitivity of 96.6%, and F-Score of 0.960, our method performs better on quality assessment metrics when compared to other existing methods. A higher F-Score is representative of a more robust algorithm with lower rates of false negatives and false positives. False positives are problematic as they may subject patients to unnecessary procedures and may reduce the availability of medical resources for others; however, false negatives are particularly detrimental as they may prevent patients from receiving medical care altogether. Our method also demonstrates superiority in the timeliness of diagnosis. With an evaluation time of 0.02 s per patient, our MiniROCKET implementation completes heart murmur detection at a much faster rate than the top models from the PhysioNet Challenge [19].

Faster algorithms provide notable advantages from both clinical and technical perspectives. Firstly, models with faster processing capabilities allow for quicker diagnostic feedback. This is particularly important in time-sensitive emergencies. Additionally, early detection of underlying cardiovascular issues allows for earlier intervention and, thus, can promote improved long-term health outcomes. Furthermore, enhanced processing allows for more patients to be screened in a shorter period of time, thus allowing increased scalability and application in medical settings. From a technical perspective, shorter training and testing times make the model easier to adapt or refine when necessary. Updating the model is necessary to enhance its accuracy and robustness, as well as to ensure it is up-to-date and effective for its assigned task. Finally, reducing training and testing times also allows for a lower computational cost, making it less resource and energy intensive. Increasing energy efficiency in this manner reduces the algorithm’s financial and environmental costs.

With recent medical and technological advancements, we have made strides in improving access to timely and accurate medical diagnosis. However, cardiovascular disease is still a critical cause for concern. Rapid and precise detection of murmurs in phonocardiogram data allows for sooner detection of underlying cardiovascular complications and thus allows early-stage treatment and improvements in long-term health outcomes. Furthermore, this method may allow for increased screening outside conventional healthcare settings, thus improving healthcare access.

However, there are certain limitations to this study. Firstly, our data had limited demographic representation, including only youth from Northeast Brazil, thus limiting generalizability to adults as well as patients of other ethnicities. Furthermore, training was conducted only when patients had recordings from all four heart valves; thus, it is unclear how the model would perform when not all recordings are present. Additionally, certain augmentation techniques, such as pitch shift and time warping, must be carefully monitored as they have the potential to create representations that deviate from physiologically normal heart function, and in doing so, affect the validity of the model’s diagnosis.

Ultimately, this paper lays a foundation for future investigations regarding the implementation of MiniROCKET in heart murmur detection and classification. Since heart murmurs can be either benign or indicative of a more serious underlying condition, one critical next step will involve the development of a robust model capable of discriminating between these two categories. Additionally, this research opens the door for further exploration into the broader application of MiniROCKET in other medical domains. For example, future studies could investigate its efficacy in the analysis of electrocardiograms or electroencephalograms for anomaly detection, potentially revolutionizing medical diagnosis.

## Figures and Tables

**Table 1:** Comparison of model performance with and without the implementation of a pre-trained Vision Transformer.

	True Positive	False Positive	True Negative	False Negative	Accuracy	Evaluation Time (s)
Without ViT	24	5	14	2	84.44%	50.405
With ViT	22	6	14	2	81.82%	0.019

**Table 2:** Comparing the performance of our model at various quantities of data augmentation.

Metric	Total Number of Spectrograms				
	928 (No Augmentation)	3712	8352	14,848	ANOVA *p*-Value
Number of Train Set Groups	185	881	2041	3665	N/A *
Number of Test Set Groups	47	47	47	47	N/A
Sensitivity	81.5% [95% CI: 77.9% to 85.2%]	95.8% [95% CI: 93.7% to 97.8%]	100% [95% CI: 100% to 100%]	100% [95% CI: 100% to 100%]	<2.00 × 10^−16^ **
Specificity	69.0% [95% CI: 66.2% to 71.9%]	87.1% [95% CI: 83.2% to 91.1%]	94.8% [95% CI: 91.0% to 98.5%]	97.6% [95% CI: 95.8% to 99.4%]	**3.79 × 10^−16^**
Positive Predictive Value	76.6% [95% CI: 74.7% to 78.4%]	90.4% [95% CI: 87.8% to 93.0%]	96.1% [95% CI: 93.4% to 98.8%]	98.1% [95% CI: 96.8% to 99.5%]	**<2.00 × 10^−16^**
Negative Predictive Value	75.4% [95% CI: 71.7% to 79.1%]	94.5% [95% CI: 92.0% to 96.9%]	100% [95% CI: 100% to 100%]	100% [95% CI: 100% to 100%]	**<2.00 × 10^−16^**
F-Score	0.789 [95% CI: 0.766 to 0.812]	0.929 [95% CI: 0.915 to 0.944]	0.980 [95% CI: 0.965 to 0.994]	0.991 [95% CI: 0.983 to 0.998]	**4.35 × 10^−16^**
Accuracy	76.0% [95% CI: 73.6% to 78.3%]	91.9% [95% CI: 90.2% to 93.6%]	97.7% [95% CI: 96.0% to 99.3%]	98.9% [95% CI: 98.1% to 99.7%]	**<2.00 × 10^−16^**
Weighted Accuracy	79.8% [95% CI: 76.7% to 82.9%]	94.6% [95% CI: 92.9% to 96.2%]	99.3% [95% CI: 98.8% to 99.8%]	99.7% [95% CI: 99.4% to 99.9%]	**<2.00 × 10^−16^**
Testing Time per Sample (seconds)	0.023	0.023	0.023	0.022	N/A

* ANOVA not conducted. ** bolded values are indicative of statistically significant *p*-values.

**Table 3:** Post Hoc Tukey Honestly Significant Difference analysis to determine significance of improvement in data augmentation.

METRIC			Sensitivity	Specificity	Positive Predictive Value	Negative Predictive Value	F-Score	Accuracy	Weighted Accuracy
Levels of Augmentation	None-Light	Mean Difference	−0.142 [95% CI: −0.178 to −0.107]	−0.180 [95% CI: −0.235 to −0.127]	0.138 [95% CI: 0.101 to 0.175]	−0.1901 [95% CI: −0.229 to −0.153]	0.190 [95% CI: 0.128 to 0.252	−0.160 [95% CI: −0.189 to −0.130]	−0.148 [95% CI: −0.178 to −0.117]
		*p*-value	<0.0001 *	**<0.0001**	**<0.0001**	**<0.0001**	**<0.0001**	**<0.0001**	**<0.0001**
	None-Medium	Mean Difference	−0.185 [95% CI: −0.220 to −0.150]	−0.257 [95% CI: −0.311 to −0.203	0.195 [95% CI: 0.158 to 0.232]	−0.246 [95% CI: −0.284 to −0.209]	0.290 [95% CI: 0.228 to 0.352]	−0.217 [95% CI: −0.246 to −0.188]	−0.195 [95% CI: −0.225 to −0.164]
		*p*-value	**<0.0001**	**<0.0001**	**<0.0001**	**<0.0001**	**<0.0001**	**<0.001**	**<0.0001**
	None-Heavy	Mean Difference	−0.185 [95% CI: −0.220 to −0.150]	−0.286 [95% CI: −0.340 to −0.232]	0.216 [95% CI: 0.179 to 0.253]	−0.246 [95% CI: −0.284 to −0.209]	0.327 [95% CI: 0.265 to 0.389]	−0.230 [95% CI: −0.259 to −0.200]	−0.199 [95% CI: −0.229 to −0.168]
		*p*-value	**<0.0001**	**<0.0001**	**<0.0001**	**<0.0001**	**<0.0001**	**<0.0001**	**<0.0001**
	Light-Medium	Mean Difference	0.042 [95% CI: 0.007 to 0.077]	0.076 [95% CI: 0.022 to 0.130]	−0.057 [95% CI: −0.094 to −0.020]	0.055 [95% CI: 0.018 to 0.093]	−0.100 [95% CI: −0.162 to −0.038]	0.057 [95% CI: 0.028 to 0.087]	0.047 [95% CI: 0.017 to 0.078]
		*p*-value	**0.012**	**0.003**	**0.001**	**0.002**	**0.0003**	**<0.0001**	**0.001**
	Light-Heavy	Mean Difference	−0.042 [95% CI: −0.078 to −0.007]	−0.105 [95% CI: −0.159 to −0.051]	0.078 [95% CI: 0.041 to 0.115]	−0.055 [95% CI: −0.093 to −0.018]	0.137 [95% CI: 0.075 to 0.199]	−0.070 [95% CI: −0.100 to −0.041]	−0.051 [95% CI: −0.081 to −0.021]
		*p*-value	**0.0124**	**<0.0001**	**<0.0001**	**0.002**	**<0.0001**	**<0.0001**	**0.0003**
	Medium-Heavy	Mean Difference	1.11 × 10^−16^ [95% CI: −0.035 to −0.035]	−0.029 [95% CI: −0.083 to 0.026]	0.021 [95% CI: −0.016 to 0.058]	0.000 [95% CI: −0.038 to 0.038]	0.038 [95% CI: −0.024 to 0.099]	−0.013 [95% CI: −0.042 to 0.017]	−0.004 [95% CI: −0.034 to 0.026]
		*p*-value	1.000	0.494	0.447	1.000	0.374	0.647	0.985

* bolded values are indicative of statistically significant *p*-values.

**Table 4:** Evaluating the model performance with heavy augmentation with a standard 80%/20% training/testing split.

Number of Train Groups	Number of Test Groups	Sensitivity	Specificity	Positive Predictive Value	Negative Predictive Value	F-Score	Accuracy	Weighted Accuracy	Time
2969	743	91.7% [95% CI: 90.4% to 92.9%]	91.7% [95% CI: 90.4% to 92.9%]	91.4% [95% CI: 90.7% to 92.1%]	91.8% [95% CI: 90.6% to 93.1%]	91.4% [95% CI: 90.7% to 92.2%]	91.5% [95% CI: 90.8% to 92.3%]	91.6% [95% CI: 90.6% to 92.7%]	0.022

**Table 5:** Comparing the performance of various models for heartbeat detection.

Reference	Method	WAcc	F-Score	Sensitivity	Evaluation Time (Seconds per Patient)
Lu et al., 2022 [18]	Lightweight Convolutional Neural Network	0.780	0.619	NR *	0.62
McDonald et al., 2022 [15]	Parallel Hidden Semi-Markov Model	0.776	0.623	NR	0.70
Xu et al., 2022 [19]	Hierarchical multi-scale Convolutional Network	0.776	0.647	NR	0.24
Walker et al., 2022 [20]	Dual Bayesian ResNet	0.771	0.686	NR	35.31
Lee et al., 2022 [21]	Light Convolutional Neural Network + ResMax	0.767	0.521	NR	1.59
Manshadi & Mihandoost, 2024 [16]	Deep Features + RFE + RF	0.930	0.910	0.910	NR
Our Model	ViT + MiniROCKET	0.916 **	**0.914**	**0.917**	**0.02**

* Not reported. ** Metrics reported by our model.

## Data Availability

The original data presented in the study are openly available in The CirCor DigiScope Dataset: From Murmur Detection to Murmur Classification at Reference [7], https://doi.org/10.1109/JBHI.2021.3137048.

## Abbreviations

The following abbreviations are used in this manuscript:

MiniROCKET	Minimally Random Convolutional Kernel Transform
ViT	Vision Transformer
STFT	Short Time Fourier Transform
